# Therapeutic efficacy of bovine ultra-long CDR H3 antibody against BVDV in challenged BALB/c mouse model

**DOI:** 10.3389/fimmu.2026.1805322

**Published:** 2026-04-07

**Authors:** Gege Rile, Qihuan Zhao, Na Qi, Bo Wang, Jingsi Mei, Hui Wang, Shujun Zhang, Fuxiang Bao

**Affiliations:** 1 College of Veterinary Medicine, Inner Mongolia Agricultural University, Huhhot, China; 2 Saikexing Breeding Biotechnology (Group) Co., Ltd., Huhhot, China; 3 Key Laboratory of Clinical Diagnosis and Treatment Techniques for Animal Disease, Ministry of Agriculture and Rural Affairs, Huhhot, China

**Keywords:** antibody purification, bovine viral diarrhea virus, neutralizing antibody, therapeutic efficacy, ultralong CDR H3 antibody

## Abstract

Bovine Viral Diarrhea Virus (BVDV), the etiologic agent of bovine viral diarrhea, poses substantial economic threats to the cattle industry worldwide. While vaccination represents the cornerstone of preventive strategies, relying primarily on inactivated whole-virus vaccines, continuous viral evolution has rendered these conventional formulations inadequately protective. Bovine ultralong CDR H3 antibodies, characterized by a 70-amino-acid composition, emerge as promising candidates for therapeutic development. However, their effectiveness against BVDV remains largely unexplored. In this study, a phage display library encoding BVDV-specific ultralong CDR H3 antibodies was successfully constructed and subjected to biopanning. Selected high-affinity antibodies were expressed and purified, and showed specific binding to BVDV with an affinity of 312.5 ng/mL and neutralizing activity (IC_50_ = 11.72 μg/mL) *in vitro*. To evaluate therapeutic efficacy *in vivo*, a BALB/c mouse challenge model was established. We found that antibody treatment significantly reduced viral load and attenuated histopathological damage compared to the control group (*P* < 0.05). These results collectively underscore the potential of ultralong CDR H3 antibody platforms as viable candidates for novel BVDV immunotherapeutics and vaccine development.

## 1 Introduction

Bovine viral diarrhea virus (BVDV), a *Pestivirus* of the *Flaviviridae* family, is the principal viral driver of the bovine respiratory disease complex. First described in the United States in 1946 (1) and isolated in 1957 (2), the virus was introduced to China in 1980 when the Changchun 184 strain was recovered from an aborted fetus in Jilin Province (3). Since then, BVDV has spread nationwide, infecting cattle, small ruminants and swine, and is now regarded as one of the most economically damaging pathogens of global livestock (4). The enveloped, single-stranded, positive-sense RNA virus is grouped into three major genotypes—BVDV-1, BVDV-2 and Hobi-like (BVDV-3)—that together encompass 26 sub-genotypes (5–8). Vaccination remains the backbone of control the infections, relying on modified-live and inactivated formulations (9); However, conventional vaccines provide only partial protection because they cannot keep pace with the virus’ high genetic diversity, rapid mutation and concurrent circulation of multiple serotypes (10). To address the insufficient cross-protection of conventional vaccines, researchers developed mosaic polypeptide chimeras that successfully induced cross-protective immune responses in calves and outperformed commercial modified live vaccines (11, 12). However, this approach is limited by complex design and lengthy validation cycles. Recently, antibody phage display technology, with its low cost and high-throughput features, has provided a new avenue for rapid development of therapeutic antibodies targeting conserved epitopes (13). Therefore, developing novel antibody immunotherapies that combine potent neutralizing activity with broad-spectrum specificity is imperative.

In therapeutic, diagnostic, and research settings, antibodies are the adaptive immune system’s workhorses, able to recognize and neutralize an almost limitless range of pathogens. This exquisite specificity stems from the hyper-diversity of the variable region, especially the complementarity-determining regions (CDRs) (14). In conventional mouse or human antibodies, the antigen-binding surface formed by the six CDRs displayed on the heavy-/light-chain heterodimer typically engages relatively flat or shallow epitopes. By contrast, the bovine antibody repertoire bifurcates into conventional (≤40 aa, ~90% frequency) and ultralong variants (50–70 aa, ~10%) (15, 16). Such ultralong CDR H3 antibodies are uniquely suited to engage recessed viral epitopes; To date, cattle are the only species shown to mount a rapid, broadly neutralizing response to the stabilized HIV-1 envelope trimer BG505.SOSIP.664 after vaccination. The ultralong CDR H3 makes contact with the buried CD4-binding site with an exceptionally small footprint (17, 18). Furthermore, the 3–6 kDa knob domain functions as a light-chain-independent antigen binder that potently neutralizes SARS-CoV-2 and greatly simplifies bispecific antibody construction (19, 20).

In this study, calves were immunized with BVDV to generate a phage display library of bovine ultralong CDR H3 antibodies, from which BVDV-specific candidates were enriched and isolated via biopanning. Selected antibodies were expressed, purified, and functionally characterized for binding and neutralizing activities. Importantly, treatment effects of the bovine ultralong CDR H3 antibody were evaluated on BVDV-infected mice, and highlighting its potential as an immunotherapeutic candidate.

## 2 Materials and methods

### 2.1 Cells and viruses

The bovine kidney cell line Madin-Darby Bovine Kidney (MDBK) and bovine rotavirus (BRV) were kindly provided by Professor Weiguang Zhou from College of Veterinary Medicine, Inner Mongolia Agricultural University. Cells were cultured in DMEM medium (Gibco, USA) supplemented with 10% (v/v) fetal bovine serum (FBS, Gibco, USA) and 1% penicillin-streptomycin at 37°C with 5% CO_2_. Bovine viral diarrhea virus (BVDV-1d-NMG, cytopathic (CP) type) was isolated and preserved in the present laboratory (21). The virus was inoculated into MDBK cell monolayers at 80%-90% confluence and incubated for 96 h at 37°C with 5% CO_2_. The stock was centrifuged at 3000 × g for 10 min at 4°C, and the supernatant was filtered through a 0.22 μm filter membrane (Merck Millipore, Burlington, MA, USA). The viral titer was determined to be 10^6.45^ TCID_50_/0.1 mL by 50% tissue culture infectious dose (TCID_50_) assay.

### 2.2 Calf immunization

A five-month-old healthy Holstein calf was purchased from a commercial dairy farm in the suburbs of Hohhot, Inner Mongolia Autonomous Region. The BVDV viral antigen stock (10^6.45^ TCID_50_/0.1 ml) was thoroughly emulsified with an equal volume of Complete Freund’s Adjuvant (CFA). This calf received a 2 mL intramuscular injection in the neck muscle (containing 1 mL viral antigen). Pre-immune serum was collected via jugular vein puncture to collect 3 ml blood prior to primary immunization, and allowed to clot at 37°C for 1–2 h, and centrifuged at 3000 × g for 10 min at 4°C. Four booster immunizations were administered on days 14, 21 and 28 post-primary immunization using Incomplete Freund’s Adjuvant (IFA)-emulsified viral antigen at the same dosage and route. Serum was harvested 7 days after each immunization, and BVDV-specific antibody titers were determined by enzyme-linked immunosorbent assay (ELISA).

The calf was maintained at the Laboratory Animal Center of the College of Veterinary Medicine, Inner Mongolia Agricultural University with free access to feed and water. All protocols complied with institutional and national animal welfare regulations and were approved by the Institutional Animal Care and Use Committee of Inner Mongolia Agricultural University (Protocol No. NND2022023).

ELISA: Microtiter plates were coated with BVDV (100 μL/well, 1:1 in coating buffer) overnight at 4°C, blocked with 3% BSA in PBST (1 h, 37°C), and incubated with serially diluted sera (1 h, 37°C) using pre-immune serum as negative control. Detection was performed using HRP-conjugated mouse anti-bovine IgG (1F2) secondary antibody (Solarbio, Beijing, China).

### 2.3 Construction of bovine ultralong CDR H3 phage display library

The phage display library was constructed as described previously with minor modifications (22). One week post final immunization, PBMCs were isolated from jugular venous blood (sodium heparin-anticoagulated) of the immunized calf using a commercial bovine lymphocyte separation kit (Haoyang Bio, Tianjin, China). Total RNA extracted with TRIzol reagent (Ambion Inc., Austin, Texas, USA) was reverse-transcribed to cDNA. Bovine ultralong CDR H3 fragments were amplified by nested PCR: first-round using primers P1/P2 (**Table 1**), followed by second-round with primers R311-R325 using the ~200 bp amplicon as template (22). The final PCR product was gel-purified after agarose gel electrophoresis.

**Table 1:** PCR primers.

Primer	Sequence
P1	5’-GGACTCGGCCACMTAYTACTG-3′
P2	5′-GCTCGAGACGGTGAYCAG-3′
R311	5′-CATGCCATGGCCACTACTGTGCACCAAAAAACA-3′
R312	5′-CATGCCATGGCCACTACTGTGCACCAAAGAACC-3′
R313	5′-CATGCCATGGCCACTACTGTGCACCAAAAAACG-3′
R314	5′-CATGCCATGGCCACTACTGTGCACCAACAAACT-3′
R315	5′-CATGCCATGGCCACTACTGTGCACCAACAGACC-3′
R316	5′-CATGCCATGGCCACTACTGTGGTCCAGAAAACA-3′
R317	5′-CATGCCATGGCCACTACTGTAGTCCAACGAACA-3′
R321	5′-AAGGAAAAAAGCGGCCGCGGCATCGACGTACCATTCGTA-3′
R322	5′-AAGGAAAAAAGCGGCCGCGGTATCGACGTACCATTCGTA-3′
R323	5′-AAGGAAAAAAGCGGCCGCGGCTTCGACGTACAATTCGTA-3′
R324	5′-AAGGAAAAAAGCGGCCGCGGCATTGACGTAGAATTCGTA-3′
R325	5′-AAGGAAAAAAGCGGCCGCGGCCTCGATGTCAAATTCGTA-3′
T7	5’-TAATACGACTCACTATAGGG-3′
T7t	5’-TGCTAGTTATTGCTCAGCGG-3′
MP57	5’-TTATGCTTCCGGCTCGTATG-3′
GIII	5’-CCACAGACAGCCCTCATAG-3′
BVDV-probe	HEX-CCTGAGTACAGGGKAG-TCGTCRGTGGTTCGAC-BHQ1
BVDV-F	5′-GAGGCTAGCCATGCCCTTAGT-3′
BVDV-R	5′-CTCGTCCACRTGGCATCTCGA-3′

Bovine ultralong CDR H3 fragments and pMECS vector (gifted by Prof. Serge Muyldermans, VUB, Belgium) were digested with *Nco*I/*Not*I (Takara Bio Inc., Shiga, Japan) and ligated using T4 DNA ligase (Takara Bio Inc., Shiga, Japan). Recombinant plasmids were electroporated into *E. coli* TG1 competent cells (GE Healthcare, Chicago, IL, USA), and transformants were selected on 2×YT agar with 2% glucose and 100 μg/mL ampicillin (37°C, 12 h). Library capacity was calculated by colony enumeration of serial dilutions. Positive clones were identified by colony PCR and further verified by DNA sequencing (primers MP57/GIII, **Table 1**) to confirm correct orientation and in-frame insertion. Glycerol stocks (25% v/v) were prepared from pooled colonies and stored at -80°C.

### 2.4 Biopanning and characterization of BVDV-specific ultralong CDR H3 antibodies from a phage display library

The antibody repertoire was subjected to three iterative selection cycles against BVDV. For phage packaging, the library was expanded 100-fold in 2×YT broth and cultured at 37°C with continuous shaking (220 rpm) prior to helper phage M13K07 (NBbiolab, Chengdu, China) infection (37°C, 2 h). Infected cells were harvested by centrifugation, resuspended in antibiotic-supplemented 2×YT-AK medium (100 μg/mL ampicillin, 70 μg/mL kanamycin), and further incubated for 12 h under the same conditions to allow phage propagation. Culture supernatants were clarified by centrifugation, and phage particles were precipitated using polyethylene glycol/NaCl (final concentration: 20% PEG8000, 2.5 M NaCl) at 4°C for 2 h. The precipitated phage pellet was collected by centrifugation and resuspended in PBS to yield the recombinant phage display library.

Immunotubes were immobilized with 3 mL BVDV overnight at 4°C. After three washes with PBST (0.05% Tween-20), tubes were blocked with 5 mL blocking buffer (5% BSA in PBST) for 1 h at 37°C. To minimize background interference during each round of selection, the ultralong CDR H3 phage library was pre-adsorbed against uninfected MDBK cell lysate-coated tubes for 30 min at 37°C, then transferred to BVDV-coated tubes and incubated for 1 h at 37°C. Bound phages were washed five times with 0.5% PBST and rescued by infection of *E. coli* TG1 for amplification and titering. Enrichment of specific binders was monitored by calculating the output-to-input phage ratio across selection rounds.

Following three rounds of biopanning, 92 clones were randomly selected for BVDV-binding assessment via Phage-ELISA. Positive hits were cultured overnight at 37°C in 400 μL 2×YT-AG (100 μg/mL ampicillin, 2% glucose). Fifty-microliter aliquots were transferred to 400 μL 2×YT-AG containing M13K07 and incubated with shaking at 37°C (220 rpm). After centrifugation, pellets were resuspended in 2×YT-AK and cultured overnight. Phage particles were harvested from the supernatant by PEG8000 precipitation and evaluated for BVDV binding using HRP-conjugated anti-M13 antibodies. Binding signals were quantified by measuring absorbance at 405 nm (OD_405_). Subsequently, Phage-ELISA-positive clones were subjected to Sanger sequencing (Sangon Biotech, Shanghai, China) and clustered based on ultralong CDR H3 amino acid sequence identity.

### 2.5 Expression and purification of bovine ultralong CDR H3 recombinant protein

Following validation by Phage-ELISA and sequencing, a recombinant positive clone plasmid bearing the bovine ultralong CDR H3 gene was ligated into the His-tagged pET-22b(+) expression vector using T4 DNA ligase. The recombinant plasmid was transformed into *E. coli* BL21(DE3) competent cells (Sangon Biotech, Shanghai, China). Upon reaching OD600≈0.6, cells were induced with 0.5 mM isopropyl-β-D-thiogalactoside (IPTG) (Solarbio Life Sciences, Beijing, China) and recombinant ultralong CDR H3 antibody protein expression was performed at 25°C for 10 h.

Bacterial cells were harvested by centrifugation (4°C, 7200×g, 10 min) and resuspended in denaturation buffer (containing 8 M urea). Following sonication (130 W, 5 s on/5 s off, 15 min total) and centrifugation (7200 × g, 10 min, 4°C), the supernatant was collected. Denatured proteins were purified by nickel ion affinity chromatography using Ni-NTA Sefinose resin (Sangon Biotech Co., Ltd., Shanghai, China) according to the manufacturer’s instructions and subjected to SDS-PAGE analysis. Refolding was performed by stepwise dialysis against decreasing urea concentrations (6 M, 4 M, 2 M, and 1 M in PBS, each for 4 h at 4°C), and followed by extensive dialysis against PBS (3 changes, 12 h total). Expression and purity were assessed by 15% reducing SDS-PAGE with Coomassie blue staining.

### 2.6 ELISA characterization of the ultralong CDR H3 antibody

Microtiter plates were coated with BVDV (100 μL/well) or irrelevant antigen BRV (100 μL/well) in coating buffer overnight at 4°C, blocked with 3% BSA in PBST (0.5% Tween-20) for 1 h at 37°C, and incubated with serially diluted purified ultralong CDR H3 antibody (1 h, 37°C). Negative control comprised purified protein from *E. coli* BL21(DE3) transformed with empty pET-22b(+) vector; PBS served as blank control. BRV-coated wells with antibody addition were used to assess binding specificity. Bound antibodies were detected using HRP-conjugated anti-6×His mouse monoclonal antibody (Proteintech Group, Wuhan, China) at 1:1000 dilution. Absorbance at 450 nm (OD_450_) was measured to evaluate binding activity.

### 2.7 Immunofluorescence

MDBK cells were seeded in 6-well plates at 10^6^ cells/well. After three PBS washes, cells were infected with BVDV (10^6.45^ TCID_50_/0.1 mL) or BRV (10^6.13^ TCID_50_/0.1 mL) in 2 mL medium; BRV-infected and uninfected wells served as irrelevant antigen and negative controls. Following 24 h incubation at 37°C (5% CO_2_), cells were washed thrice with PBS, and fixed with 4% paraformaldehyde for 20 min at RT, and blocked with 5% BSA for 1 h at RT. Ultralong CDR H3 antibody (50 μg/mL) was incubated overnight at 4°C. After three 5 min PBS washes, cells were stained with CoraLite^®^488-conjugated anti-6×His mouse monoclonal antibody (1:500 dilution; Proteintech Group, Wuhan, China) for 1 h at RT protected from light. Nuclei were counterstained with 200 μL DAPI for 10 min at RT. Following three final PBS washes, fluorescent images were captured using an inverted fluorescence microscope.

### 2.8 *In vitro* neutralization assay

MDBK cells were seeded in 96-well plates at 2×10^5^ cells/mL (100 μL/well) and incubated for 12 h at 37°C (5% CO_2_). Ultralong CDR H3 antibody was serially diluted (40, 20, 10, and 5 μg/mL) in PBS. Each dilution (100 μL) was mixed with equal volume of BVDV (10^6.45^ TCID_50_/0.1 mL) and incubated at 37°C for 1 h. The antibody-virus mixture was added to cells and incubated for 72–96 h at 37°C (5% CO_2_). Virus-only and mock-infected controls were included. Cell viability was assessed using 10 μL CCK-8 reagent (Solarbio, Beijing, China). as per the manufacturer’s protocol, and absorbance at 450 nm (OD_450_) was measured.

### 2.9 Therapeutic application of bovine ultralong CDR H3 antibody on BVDV challenged mice

Fifteen Specific-pathogen-free (SPF) BALB/c mice (6–8 weeks old, 18–22 g) were purchased from Henan Skybio Biotechnology Co., Ltd. (Henan, China). All mice were housed in Laboratory Animal Center of the College of Veterinary Medicine, Inner Mongolia Agricultural University with ad libitum access to food and water. Experimental procedures were conducted in strict accordance with the guidelines approved by the Institutional Animal Care and Use Committee of Inner Mongolia Agricultural University (Protocol No.: NND2023024).

Mice were randomly allocated into three groups (n=5): (1) BVDV infection, (2) CDR H3 antibody treatment, and (3) negative control. Mice were challenged intraperitoneal injection with 0.4 mL BVDV (10^6.45^ TCID_50_/0.1 mL) or DMEM (group 3) (23, 24). At 24 h post-infection, group 2 received 0.2 mL ultralong CDR H3 antibody (1 mg/kg) through tail intravenous injection, while groups 1 and 3 received an equal volume of PBS. Fecal samples were collected on days -1, 1, 3, 5, 7, 10, and 14 post-infection. On days 7, one mouse per group was euthanized by cervical dislocation for tissue harvest (liver, spleen, duodenum, jejunum). Therapeutic efficacy was evaluated by clinical scoring, virological analysis, and histopathology.

### 2.10 Viral RNA detection by RT-PCR

Viral genomic RNA was extracted from mouse feces using a column-based viral genomic DNA/RNA extraction kit (Tiangen Biotech, Beijing, China). RT-PCR amplification was performed using virus-specific primers BVDV-F and BVDV-R (**Table 1**) targeting the BVDV 5′ untranslated region (5′-UTR).

### 2.11 Real-time quantitative reverse transcription PCR

The One Step Prime Script RT-PCR Kit (Takara Bio Inc., Shiga, Japan) was used to amplify the BVDV 5′-UTR and clone into the pMD19-T vector to generating recombinant plasmid pMD19-T-5′-UTR. This plasmid was prepared by 10-fold serial dilution from 10^9^ copies/μL to 10^1^ copies/μL with each concentration run in triplicate for standard curve construction.

Thermal cycling was performed on a Bio-Rad CFX96 system in 25 μL reactions: 42°C 5 min (RT), 95°C 10 s, followed by 45 cycles of 95°C 5 s and 60°C 30 s, with melting curve analysis. The standard curve (10^9–^10^1^ copies) data were analyzed using GraphPad Prism 10, yielding R^2^>0.99 and 95% amplification efficiency. Viral copy numbers were derived by interpolating sample Cq values against this regression.

### 2.12 Histopathological examination

On day 7 post-infection, liver, spleen, duodenum, and jejunum were harvested from one mouse per group and fixed in 10% neutral formalin. Fixed tissues were processed through graded ethanol dehydration, xylene clearing, and paraffin infiltration before embedding. Paraffin blocks were sectioned at 4-6 μm using a rotary microtome (Kedi KD-202A, Hangzhou, China). Sections were floated on a 37°C-40°C water bath to flatten, and mounted on slides and dried at 60°C for 30 min to cool to RT, and stained with hematoxylin and eosin (H&E). Slides were examined under a light microscope.

### 2.13 Statistical analysis

Data are expressed as mean ± standard deviation (x ± SD; bar height, mean; error bars, SD). Statistical analyses were performed using GraphPad Prism 10.0 (GraphPad Software, La Jolla, CA, USA). A P-value less than 0.05 was considered statistically significant. In the figures, statistical significance is indicated as follows: *** *P* < 0.001; ** *P* < 0.01; * *P* < 0.05; ns: no significance. *In vitro* experiments were performed in triplicate. For the mouse challenge model, one experiment was performed with mice randomly divided into three groups (n=5 per group).

## 3 Results

### 3.1 ELISA determination of serum antibody response in BVDV-immunized calf

**Figure 1:**
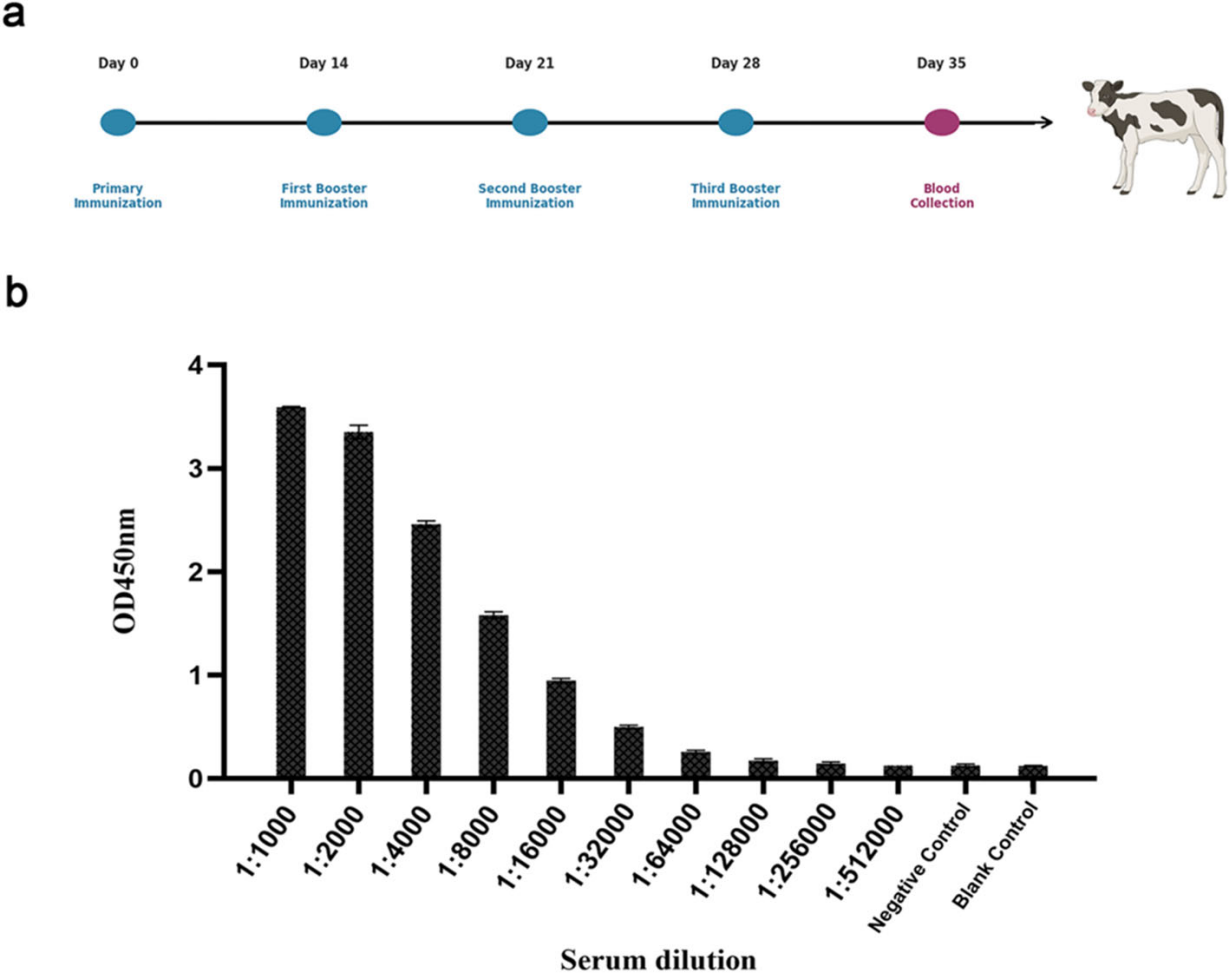
ELISA analysis of BVDV-specific antibody titers in immunized calf sera. **(a)** Schematic diagram of the immunization and sampling protocol. **(b)** X-axis, serum dilution fold; Y-axis, optical density at 450 nm (OD_450_).

To evaluate the immune response of calves to BVDV, the BVDV viral antigen was emulsified with Freund’s adjuvant and administered intramuscularly to a Holstein calf, followed by three booster immunizations.The immunization and blood collection schedule of the calf is shown in **Figure 1a**. Seven days after the final immunization, serum samples collected pre− and post−immunization were analyzed by ELISA. As shown in **Figure 1b**, while the pre-immune serum showed an OD_450_ of 0.126, post-immunization serum remained positive (OD_450_ > 2.1-fold above negative control) even at a 1:32000 dilution, demonstrating a strong humoral immune response.

### 3.2 Construction of bovine ultralong CDR H3 phage library

**Figure 2:**
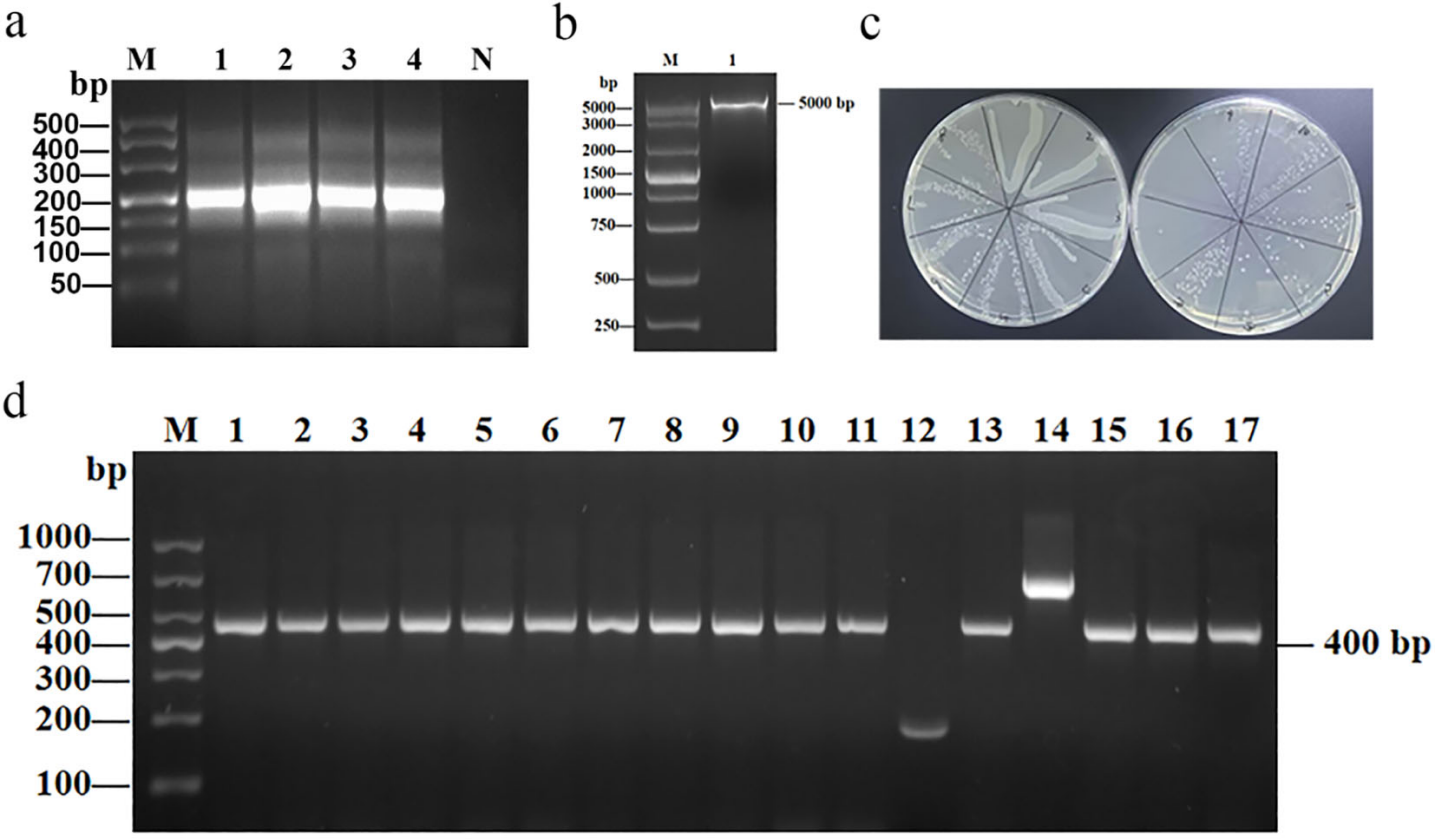
Construction of ultralong CDR H3 antibody library and screening/identification of positive clones. **(a)** PCR amplification of the bovine ultralong CDR H3 gene. M: DNA Marker; 1-4: PCR amplification products (1-2: first round, 3-4: second round), target bands were both ~200 bp; N: negative control. **(b)** Verification of pMECS plasmid by restriction enzyme digestion. M: DNA marker; 1: digested plasmid fragment ~5000 bp. **(c)** Plate culture showing 10-fold serial dilutions for titer determination of the ultralong CDR H3 phage antibody library. **(d)** PCR screening of recombinant antibody clones. M: DNA marker; lanes 1-17: randomly picked colonies; positive clones show ~400 bp insert.

To construct the bovine ultralong CDR H3 phage display library, we first extracted the total RNA from peripheral blood lymphocytes of the immunized calf and reverse-transcribed it into cDNA. As a result, the expected size (~200 bp) for ultralong CDR H3 fragments was successfully amplified by nested PCR. (**Figure 2a**). These purified fragments were then ligated into the digested pMECS plasmid (**Figure 2b**) and electroporated into *E. coli* TG1 competent cells. As shown in **Figure 2c**. A BVDV-specific phage library was successfully constructed with a titer of 2.3×10^12^ pfu. To further detect the gene insertion rate of the pMECS-CDR H3 recombinant plasmid, seventeen single colonies were selected for PCR screening. As a result, fifteen colonies (88.2%) yielded amplicons of the expected size (~400 bp) for ultralong CDR H3 inserts (**Figure 2d**). Sequencing analysis of positive clones revealed distinct CDR sequences, confirming the constructed library exhibited rich sequence diversity.

### 3.3 Phage-ELISA screening of BVDV-specific ultralong CDR H3 antibody

**Figure 3:**
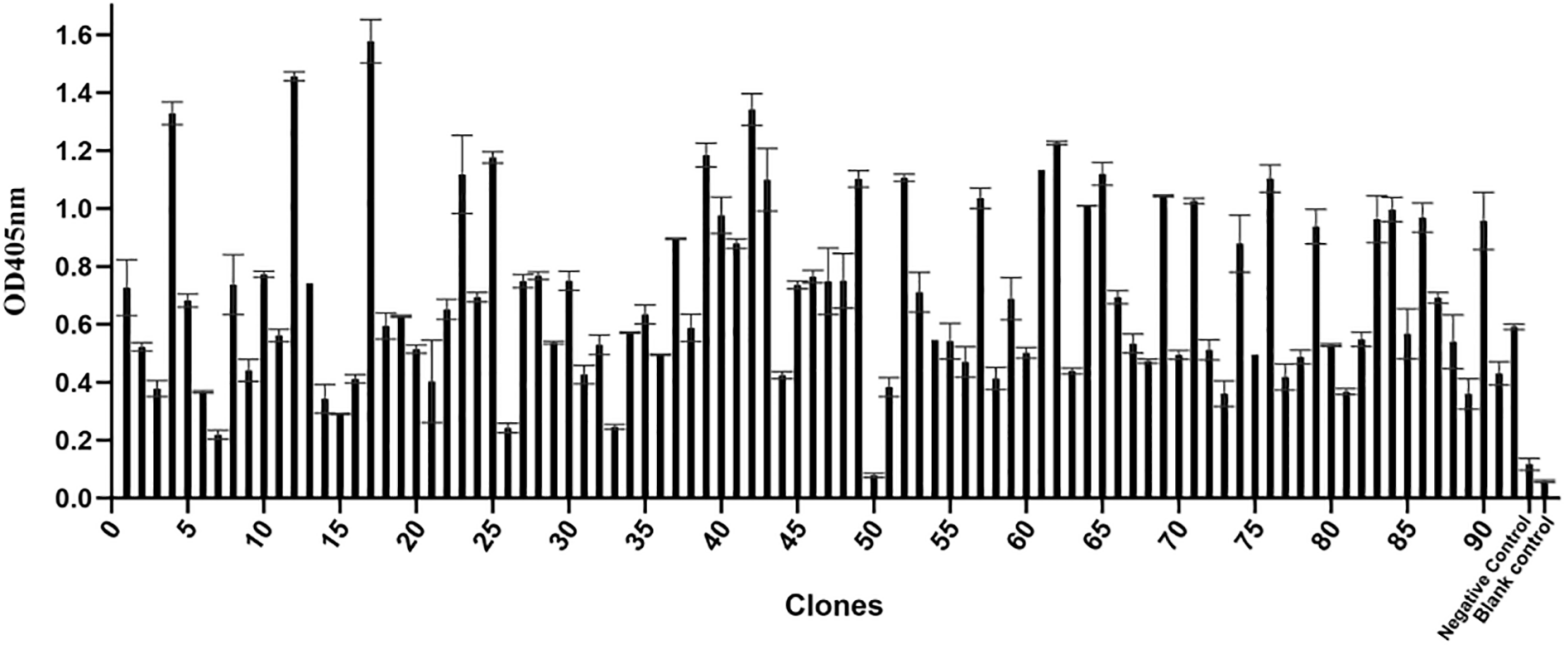
Phage-ELISA analysis of colonies. X-axis: clone numbers; Y-axis: absorbance at 405 nm (OD_405_). Error bars represent standard deviations of replicate wells. Negative control: Helper phage M13K07; Blank control: PBS.

To screen for BVDV-specific ultralong CDR H3 antibodies, three rounds of biopanning were conducted, and the enrichment fold of specific phage clones reached 430-fold in the third round, indicating efficient enrichment of target phage clones (**Supplementary Table 1**). Ninety-two individual clones were then randomly selected from the enriched library and screened for binding to BVDV via phage-ELISA, using PBS as blank control and helper phage M13K07 as negative control. As a result, the negative control exhibited an OD_405_ of 0.116, which defined a positive threshold of OD_405_ >0.336. (**Figure 3**). Based on this criterion and subsequent sequence verification, clone 17 was selected for further study.

### 3.4 Expression, purification and functional characterization of a BVDV-specific bovine ultralong CDR H3 antibody

**Figure 4:**
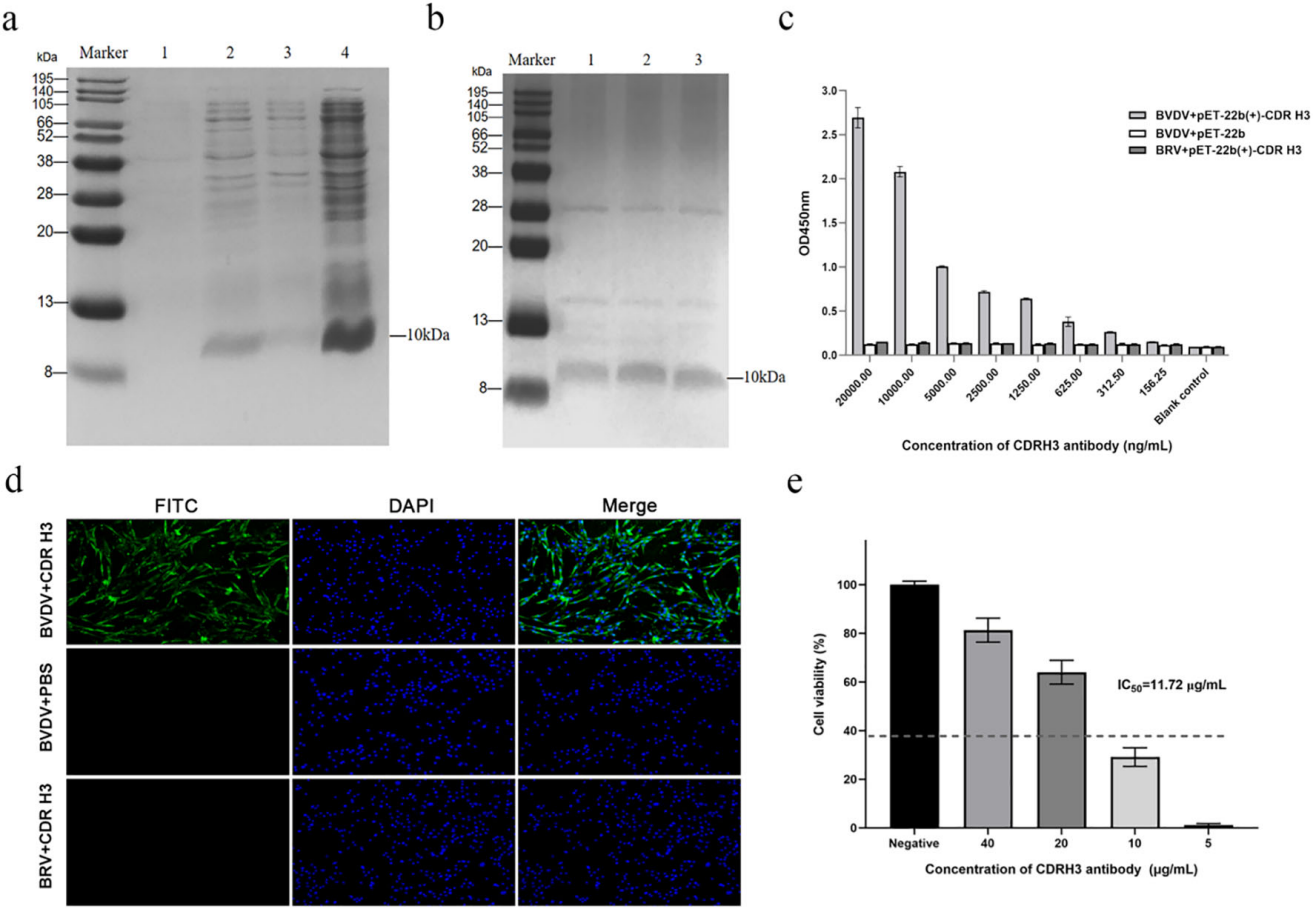
Expression, identification, and antiviral activity analysis of recombinant ultralong CDR H3 antibody. **(a)** SDS-PAGE analysis of recombinant ultralong CDR H3 antibody induced expression. Marker, protein marker; 1-4: uninduced culture, induced culture, lysate supernatant, and pellet respectively; arrow, target band (~10 kDa). **(b)** Purified and renatured recombinant ultralong CDR H3 antibody. Marker, protein marker; 1-3: renatured antibody; arrow, target protein (~10 kDa). Higher molecular weight contaminants, a typical artifact of nickel ion-chelate chromatography, are also visible. **(c)** Titer determination of recombinant ultralong CDR H3 antibody by ELISA. **(d)** Immunofluorescence assay of recombinant ultralong CDR H3 antibody binding to BVDV-infected MDBK cells. Green fluorescence (FITC) indicates antibody binding; blue fluorescence (DAPI) indicates nuclei; Merge shows overlay images. **(e)***In vitro* neutralization assay of recombinant ultralong CDR H3 antibody against BVDV. The half-maximal inhibitory concentration (IC_50_) is 11.72 μg/mL.

To verify that the phage-selected ultralong CDR H3 antibody retains high affinity and neutralizing activity against BVDV, we carried out prokaryotic expression, purification and a panel of *in vitro* functional assays. For expression of this antibody, the bovine ultralong CDR H3 gene was cloned into the His-tagged pET-22b(+) vector. The resulting plasmid was transformed into *E. coli* BL21(DE3) competent cells, and positive clones were identified by colony PCR (**Supplementary Figure 1**). Following induction and purification, SDS-PAGE analysis revealed a single band at approximately 10 kDa, consistent with the expected size of the antibody fragment (**Figures 4a, b**). Subsequently, the affinity of the BVDV-specific bovine ultralong CDR H3 antibody was examined by ELISA. As depicted in **Figure 4c**, the bovine ultralong CDR H3 antibody showed specific binding to BVDV at 312.5 ng/mL with absorbance *>*2.1-fold that of the negative control (OD_450_ = 0.125), whereas no significant binding was observed to the irrelevant antigen BRV (OD450 < 0.05), confirming the specificity of the antibody. Consistently, immunofluorescence staining confirmed specific recognition of BVDV-infected MDBK cells by the antibody (**Figure 4d**), with no detectable binding to BRV-infected cells, further demonstrating its specificity for BVDV. In the neutralization assay, CCK-8 analysis gave an IC_50_ of 11.72 μg/mL and dose-dependent protection from 5 to 40 µg/mL, demonstrating potent *in vitro* neutralizing activity (**Figure 4e**). Collectively, these data confirm that the phage-selected ultralong CDR H3 antibody was efficiently expressed and purified, and possesses both specific binding capacity and significant neutralizing activity against BVDV.

### 3.5 Ultralong CDR H3 antibody reduces viral shedding in BVDV-infected mice

**Figure 5:**
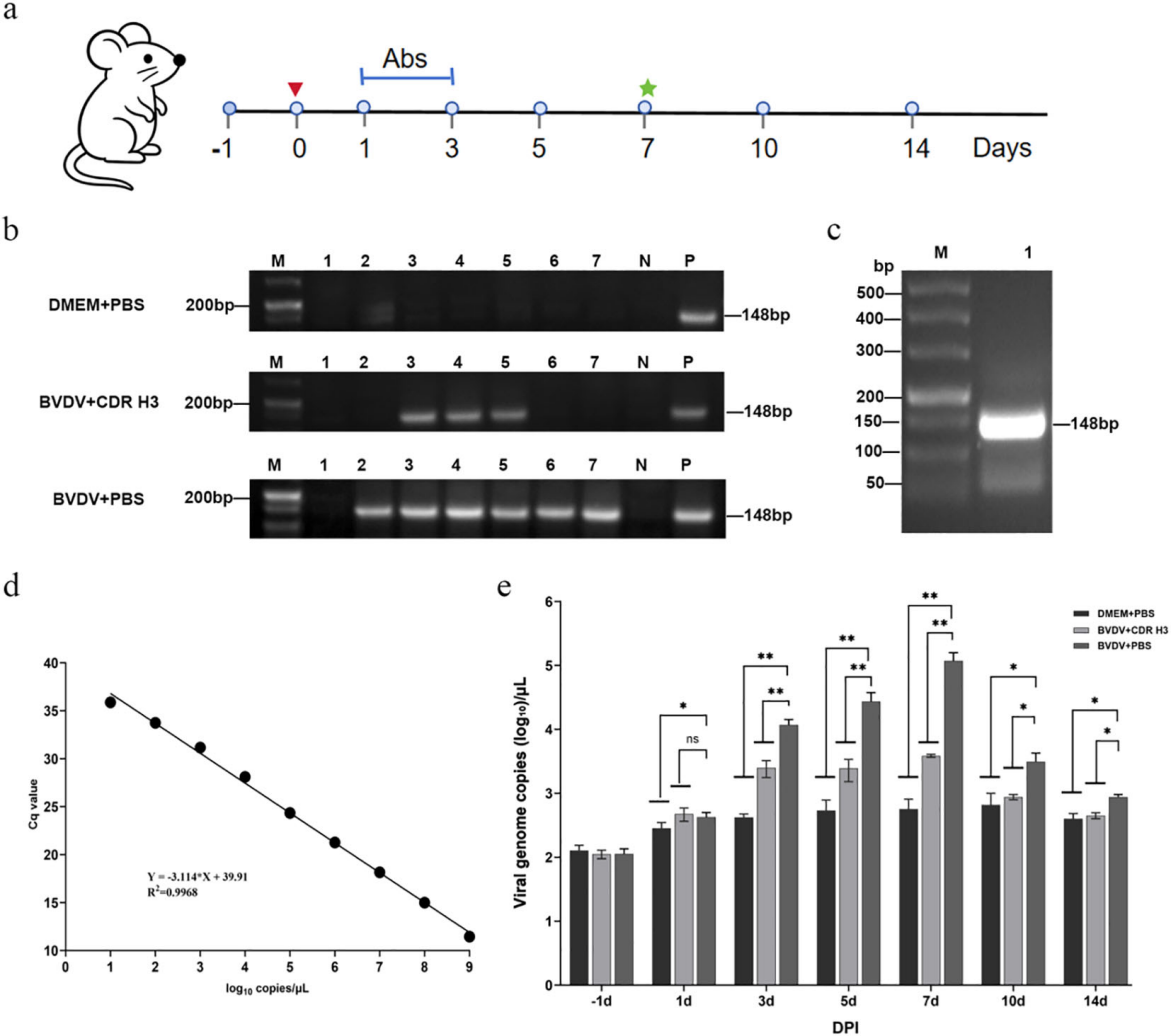
Therapeutic efficacy of ultralong CDR H3 antibodies in BVDV-infected mice. **(a)** Schematic of the experimental design. Red arrow: time of BVDV infection (day 0); Ab: antibody treatment period (days 1–3, once daily); green symbols: time points for euthanasia and sample collection. **(b)** RT-PCR detection of BVDV RNA in murine samples.M, DNA molecular-weight marker; lanes 1–7, specimens collected at the indicated time points post-challenge; P, BVDV-positive control; N, PBS-negative control. **(c)** RT-PCR amplification of the BVDV 5′-UTR gene. M: DNA marker; lane 1: target band (148 bp). **(d)** RT-qPCR standard curve. The fitted equation is Y = −3.114X + 39.91, R^2^ = 0.9948. **(e)** Comparison of viral loads at different time points. Viral genome copies were quantified by RT-qPCR and are presented as log_10_ copies/µL. The limit of detection (LOD) was 2.22 log_10_ copies/µL (Cq = 33.0), representing the lowest reliably detectable level with 95% detection rate. Days 1–7 (n = 5); days 10, 14 (n = 4). Data are presented as mean ± SD. Statistical significance is indicated by *P < 0.05, **P < 0.01; “ns” indicates no significant difference.

To evaluate the therapeutic efficacy of recombinant bovine ultralong CDR H3 antibody *in vivo*, a BALB/c mouse model of BVDV infection was established. Mice challenged intraperitoneally (i.p.) with BVDV remained asymptomatic throughout the experiment, with stable body temperature and gradually increasing body weights without significant intergroup differences (*P* > 0.05, **Supplementary Figure 2**). The timeline was shown in **Figure 5a**, mice were challenged i.p. with 0.4 mL BVDV (10^6.45^ TCID_50_/0.1 mL), or DMEM as control. At 24 h post-infection, BVDV-infected mice were treated tail intravenous injection (i.v.) with 0.2 mL of ultralong CDR H3 antibodies (1 mg/kg, once daily for 3 consecutive days), while the control group received an equal volume of PBS via the same route. Faecal samples were collected on days −1, 1, 3, 5, 7, 10 and 14 post-infection (p.i.), and viral RNA was extracted for RT-PCR and RT-qPCR analyses. As a result, the DMEM+PBS group was consistently BVDV-negative, confirming absence of virus. In the BVDV + CDR H3 antibody group, specific amplicons were detected only at 1–3 days p.i., while bands disappeared from day 5 onwards, indicating antibody-mediated suppression of viral replication. In contrast, the BVDV+PBS group showed the BVDV-specific gene fragment at every time point (**Figure 5b**).

For quantitative analysis, a 148 bp fragment of the BVDV 5′-UTR was amplified (**Figure 5c**) and cloned into the pMD19-T vector. Positive clones were subsequently identified by colony PCR (**Supplementary Figure 3**) and verified by sequencing. A ten-fold dilution series of the resulting pMD19-T-5′-UTR recombinant plasmid (10^9–^10^1^ copies/μL) was used to generate a standard curve for one-step RT-qPCR. The linear regression equation was Y = −3.114X + 39.91 (X = log_10_ RNA copies, Y = mean Cq), with R^2^ = 0.9968, demonstrating excellent linearity across the dynamic range (**Figure 5d**). Viral loads in faecal samples were then quantified using this curve. As a result, viral loads in the BVDV + CDR H3 antibody group were significantly lower than those in the BVDV+PBS group at all time points (*P* < 0.05), differences were highly significant on days 3, 5 and 7 (*P* < 0.01) and remained significant on days 10 and 14 (*P* < 0.05). These results demonstrate that the ultralong CDR H3 antibody confers potent therapeutic activity against BVDV infection in mice (**Figure 5e**).

### 3.6 Pathological tissue changes in mice after treatment with ultralong CDR H3 antibodies

**Figure 6:**
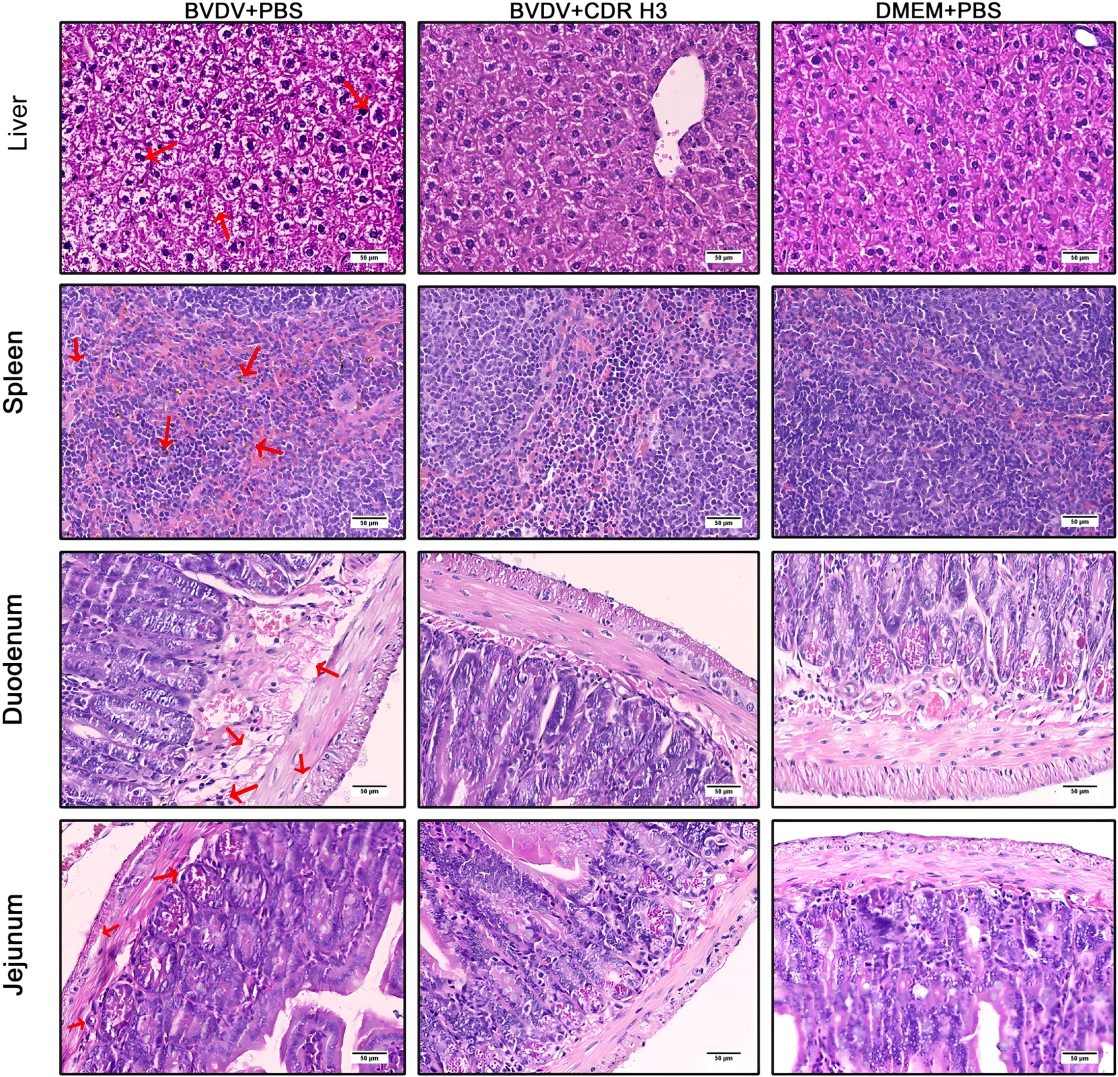
Histopathology of spleen, liver, duodenum and jejunum on day 7 post-infection. BVDV+PBS group: lesions including splenic red-pulp hemorrhage and hemosiderin deposition, hepatocyte swelling with vacuolation and necrosis, duodenal epithelial necrosis and inflammatory infiltration, and jejunal villous necrosis with lost architecture. BVDV+CDR H3 group: largely preserved histo-architecture with minimal or no abnormalities. DMEM+PBS group: entirely normal histological appearance. Hematoxylin and eosin; scale bar = 50 μm.

On day 7 post-infection, spleen, liver, duodenum and jejunum were collected for histopathological examination. As shown in **Figure 6**, mice in the BVDV-challenge control group (BVDV+PBS) showed hepatic lesions: prominent hepatocyte swelling, extensive vacuolar degeneration and multifocal necrosis. In the spleen, red-pulp hemorrhage, numerous hemosiderin-laden macrophages, lymphoid hyperplasia and indistinct red/white pulp borders were noted. The Duodenal mucosal epithelium exhibited moderate degeneration, necrosis and sloughing, accompanied by marked Lamina-propria edema and inflammatory cell infiltration. Similar changes—epithelial necrosis and sloughing, blurred demarcation between Lamina propria and Submucosa, and pronounced inflammatory cell infiltration—were seen in the Jejunum. By contrast, antibody-treated mice (BVDV+CDR H3) displayed significantly alleviated changes: Liver architecture was largely intact with only mild cytoplasmic vacuolation and no necrotic foci, Spleen appeared normal, devoid of hemosiderin deposition, congestion or lymphoid hyperplasia. Intestinal mucosa was intact with regular villous morphology, minimal or absent inflammatory infiltration, and clear Lamina-propria boundaries. All organs from mice of negative-control group (DMEM+PBS) showed completely normal histology.

## 4 Discussion

BVDV poses an enduring threat to the global cattle industry inducing reproductive failure, immunosuppression and elevated susceptibility to secondary infections, which collectively incur substantial economic losses worldwide. Line of evidence shows that live-attenuated and inactivated vaccines still dominate BVDV prophylaxis, but safety concerns and suboptimal immunogenicity restrict their clinical utility (25). The development of potent and BVDV-specific neutralizing antibodies represents a pivotal strategy for the prevention and control of BVDV infection. Bovine ultralong CDR H3 antibodies, featured by their unique structural architecture and extraordinary diversity, are ideal candidate molecules for antibody-based therapeutics and novel targeting tools (26). In this study, we constructed a highly diverse phage-display library of bovine ultralong CDR H3 antibody fragments, followed by screening and isolation of a BVDV-specific ultralong CDR H3 antibody. We further performed a systematic validation of its functional activity *in vitro* and evaluated its therapeutic efficacy in a BVDV-challenged BALB/c mouse model. These findings provide novel theoretical insights and experimental evidence for the development of next-generation antibody therapeutics against BVDV.

Phage display technology has been applied for the efficient enrichment and screening of bovine ultralong CDR H3 antibodies against a variety of antigens (22, 27). The present study further demonstrated that this technology is also applicable to enveloped viruses such as BVDV, and high-affinity positive clones targeting BVDV were successfully obtained through three rounds of biopanning. Notably, although the antibodies obtained in this study existed as inclusion bodies in the prokaryotic expression system, they could restore the correct spatial folding after *in vitro* refolding and retain favorable neutralizing activity. This is consistent with the literature report that ultralong CDR H3 antibodies can achieve functional expression in *E. coli (*22), thus avoiding the cumbersomeness and high cost associated with mammalian cell expression. It not only expands the application scope of this special type of antibody in the field of veterinary enveloped viruses, but also significantly lowers the research threshold for bovine ultralong CDR H3 antibodies, and provides a reproducible technical route for the rapid antibody-based intervention against bovine enveloped viruses.

Bovine ultralong CDR H3 antibodies display a characteristic “stalk-knob” architecture (≈70 residues forming a rigid ≈45 Å protrusion) that penetrates glycan-shielded epitopes inaccessible to conventional antibodies, endowing exceptionally potent and broad neutralization across divergent viral models (20, 28). It has been reported that the bovine antibody NC-Cow1 against HIV achieves 72% breadth (84/117 isolates) against a globally representative pseudovirus panel, with a median IC_50_ of 0.028 µg/mL, positioning it among the most potent HIV-1 broadly neutralizing antibodies described to date (18, 25). For SARS-CoV-2, the human antibody CR3022-M targets a cryptic RBD epitope and neutralizes authentic virus with an IC_50_ of 0.35 µg/mL (29). Moreover, bovine hyper-immune IgG confers complete protection and markedly reduces pulmonary and nasal viral loads in mouse challenge models, suggesting that ultralong CDR H3 antibodies exploit conserved receptor-binding-site motifs to mediate cross-neutralization (30). Neutralizing antibodies targeting BVDV have been generated in previous studies. For instance, among the 12 anti-BVDV monoclonal antibodies, 10 exhibited *in vitro* neutralizing activity against the BVDV-1a NADL strain, with neutralizing activity ranging from <69.33 nM (BVD/CA1, BVD/CA3, and BVD/CA73) to 1, 670 nM (BVD/PX14), while two monoclonal antibodies, BVD/PX18 and BVD/CA82, showed no detectable neutralizing activity. Among these antibodies, only the BVD/CA34 monoclonal antibody could recognize both BVDV-2 and BVDV-1 strains (31). In addition, VHH antibodies targeting the E2 protein significantly reduced viral RNA copy numbers in MDBK cells at a concentration of 100 μg/mL (32). In this study, the ultralong CDR H3 antibody exhibited notable neutralizing against the BVDV-1d-NMG strain at a concentration of 11.72 µg/mL, demonstrating the efficacy of this ultralong CDR H3 scaffold, and its favorable potency at a relatively low concentration represents a prominent characteristic of the antibody described herein.

To evaluate the *in vivo* therapeutic efficacy of the antibody, we established a BVDV–BALB/c mouse model by intraperitoneal inoculation. Although the mice remained clinically asymptomatic—consistent with previous reports (23, 33, 34), both viral RNA detection and histopathology confirmed successful infection, validating the model’s utility for assessing interventions independent of clinical signs. In this model, administration of the bovine ultralong CDR H3 antibody significantly reduced fecal viral shedding and attenuated tissue damage, which suggesting that its antiviral activity does not depend on symptom alleviation but may operate through direct suppression of viral replication or modulation of early innate immune responses. Notably, although BVDV can spread through blood to infect reproductive organs in cattle, we did not examine mouse uterus in this study. Previous studies showed that BVDV in mice infects lymphoid tissues (spleen, bone marrow, lymph nodes, Peyer’s patches), with no mention of reproductive organs including uterus (24, 35, 36). Therefore, we focused on tissues where BVDV is known to cause pathology. Future studies employing ELISA−based competition assays, immunohistochemical analysis and structural analysis of antigen−antibody complexes will help delineate the precise binding epitope, tissue distribution characteristics and guide rational antibody engineering. Furthermore, since murine immune responses differ from those of the natural bovine host, the therapeutic efficacy of this antibody should be re−evaluated in bovine primary cells and in cattle under field conditions. Broad neutralizing activity is unclear: Only a single BVDV-1d-NMG strain was tested in this study, whereas multiple genotypes including BVDV-1a through 1u and BVDV-2 circulate in cattle; cross-neutralization assays are required to validate its broad-spectrum efficacy.

## 5 Conclusions

In summary, we successfully constructed a BVDV-specific bovine ultralong CDR H3 phage display library. High-affinity target antibodies were obtained through biopanning, induction expression, and purification. Therapeutic evaluation using a BVDV-BALB/c mouse infection model demonstrated that this bovine ultralong CDR H3 antibody effectively inhibited BVDV infection *in vivo*, and reduced tissue damage in mice. These results provide a theoretical basis for future clinical trials in cattle and the development of novel anti-BVDV therapeutic agents.

## Data Availability

The original contributions presented in the study are included in the article/**Supplementary Material**. Further inquiries can be directed to the corresponding authors.
