# Supplementary material for: Therapeutic efficacy of bovine ultra-long CDR H3 antibody against BVDV in challenged BALB/c mouse model
Source: Front Immunol. 2026 Apr 7;17:1805322. doi: 10.3389/fimmu.2026.1805322 (PMC13095820; doi:10.3389/fimmu.2026.1805322)
Supplement: Supplementary file 1 [file Supplementaryfile1.pdf]

Supplementary Material

1 Supplementary Figures and Tables

1.1 Supplementary Tables

Panning times	Input	Output	Enriching factor (input/output)
1	1.6×10^{11}	4.0×10^{8}	4.0×10^{2}
2	9.6×10^{10}	1.2×10^{6}	8.0×10^{4}
3	8.6×10^{8}	5.0×10^{3}	1.72×10^{5}

Supplementary Table 1. Enrichment of BVDV-specific phages during three rounds of bio-panning. Bovine ultralong CDRH3 antibodies targeting BVDV were selected through three consecutive rounds of phage bio-panning. Significant enrichment of BVDV-specific binding phages was observed throughout the panning process.

1.2 Supplementary Figures

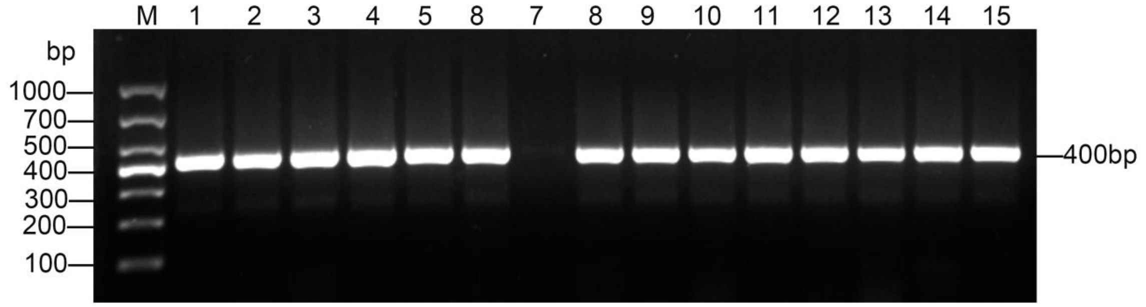

Supplementary Figure 1. Colony PCR identification of pET-22b(+)-CDR H3 recombinant plasmids. The bovine ultra-long CDRH3 gene was digested with NcoI/NotI and ligated into the pET-22b(+) vector to generate the recombinant plasmid pET-22b(+)-CDR H3. The plasmid was transformed into *E. coli* BL21(DE3) competent cells, and single colonies were randomly selected for colony PCR identification. In the figure, M represents the DNA marker, and lanes 1-15 show the screening results of positive clones.Colony PCR yielded a specific band of approximately 400 bp, indicating the successful construction of the pET-22b(+)-CDR H3 recombinant plasmid.

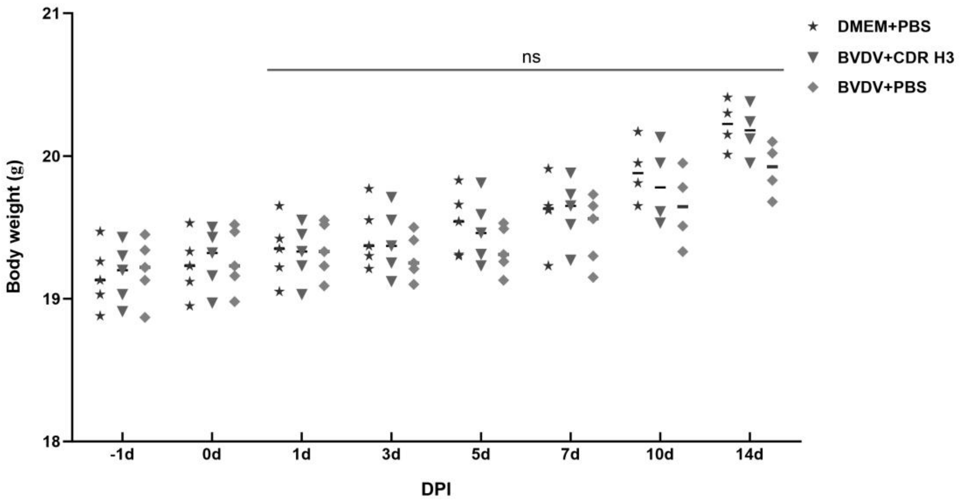

Supplementary Figure 2. Body weight changes in BALB/c mice during 14-day observation. Data are presented as mean ± SD. No significant differences were detected among DMEM+PBS, BVDV+PBS, and BVDV+CDR H3 groups (one-way ANOVA, $P > 0.05$).

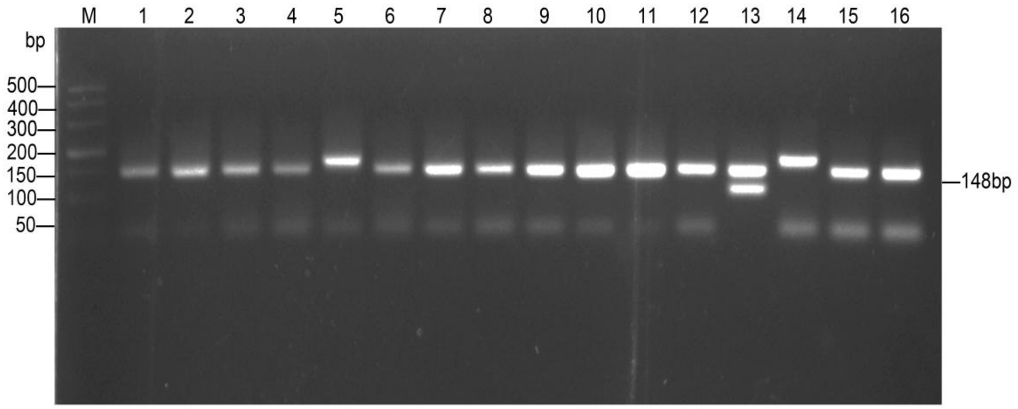

Supplementary Figure 3. Colony PCR identification of pMD19-T-5′-UTR recombinant plasmids. The 5′-UTR fragment of BVDV was cloned into pMD19-T vector and transformed into *E. coli* DH5α competent cells. Single colonies were randomly selected for colony PCR screening. Lane M, DNA marker; lanes 1–16, PCR products from individual colonies. The specific band of 148 bp (consistent with the expected size) indicates successful insertion of the 5′-UTR fragment. Positive clones were verified by sequencing to confirm correct insertion.
